# Are practice nurse-led patient consultations acceptable for the general population in Germany? Results from a population-based survey

**DOI:** 10.1080/13814788.2026.2716980

**Published:** 2026-08-27

**Authors:** Celina Wiens, Felix Lässig, Thomas Frese, Marcus Heise, Jan Schildmann, Rafael Mikolajczyk, Solveig Weise

**Affiliations:** a Institute of General Practice and Family Medicine, Interdisciplinary Centre for Health Sciences, Medical Faculty of the Martin Luther University Halle-Wittenberg, Halle (Saale), Germany; b Department of Obstetrics and Gynecology, Medical faculty of the Martin Luther University Halle-Wittenberg, Halle (Saale), Germany; c Institute for History and Ethics of Medicine, Interdisciplinary Centre for Health Sciences, Medical Faculty of the Martin Luther University Halle-Wittenberg, Halle (Saale), Germany; d Institute for Medical Epidemiology, Biometrics and Informatics, Interdisciplinary Centre for Health Sciences, Medical Faculty of the Martin Luther University Halle-Wittenberg, Halle (Saale), Germany

**Keywords:** Primary care nursing, general practice*, primary health care/organisation & administration*, professional role*, advanced practice nursing*

## Abstract

**Background:**

Delegating tasks from the general practitioners (GPs) to practice nurses (PNs) may support healthcare challenges. While PN-led care is well accepted in countries with established models, transferability to Germany remains unclear. Qualitative data suggest openness towards PN-led consultations (PNLC), but quantitative evidence is scarce.

**Objectives:**

To assess willingness, condition-specific preferences, and influencing factors towards PNLC in general population.

**Methods:**

We conducted a cross-sectional online survey within the HeReCa-Study panel between July to September 2023 using a pretested questionnaire. Participants were randomly selected from five German states, stratified by urbanity and age. Analyses included descriptive statistics, bivariate and multivariable logistic regression (IBM SPSS 25.0; R 4.5.2).

**Results:**

The response was 50.0% (1,532/3,066). Approximately 39.7% received care in single-handed practices (SHP). Among included participants without prior PNLC experience (*n* = 1,419), willingness to accept PNLC was 83.6%. Willingness was highest for superficial wounds, respiratory infections, and chronic conditions and lowest for new-onset back pain. Higher trust in PNs’ professional (OR 2.46; 95% CI 1.60–3.82) and communication skills (OR 1.80; 1.18–2.75) increased willingness. Older age (OR 0.32; 0.10–0.90 in 74–83 versus 22–33-year-olds) and treatment in SHP vs. no (OR 0.69; 0.48–0.99) were associated with lower willingness. Sex, regional variables and population density showed no relevant associations.

**Conclusion:**

Acceptability of PNLC among the German general population is high. Trust in PNs’ professional and communication skills, and younger age, are associated with increased willingness for PNLC, while treatment in SHP showed a negative association. Our findings may inform implementation of PNLC in other countries.

## KEY MESSAGES

- Adults from the general population showed high acceptance of practice nurse-led consultations (PNLC) for conditions such as arterial hypertension, diabetes mellitus and acute respiratory tract infections.
- Participants’ trust in practice nurse’s increased PNLC acceptance.
- PNLC should be initially introduced in group practices and younger populations.

**POINT OF INTEREST:**

- Adults from the general population showed high acceptance of practice nurse-led consultations (PNLC) for conditions such as arterial hypertension, diabetes mellitus and acute respiratory tract infections.
- Participants’ trust in practice nurses increased acceptance of PNLC.
- Older participants and those attending single-handed practices were less open to PNLC.

## Introduction

Demographic change is increasing the proportion of elderly patients with multimorbidity while reducing the availability of qualified healthcare professionals [1–3]. One-third of European general practitioners (GPs) are aged 55 or older [1]. In Germany, around 11,000 GP positions are expected to remain vacant in the coming years, threatening care in 40% of districts [4]. Task shifting from GPs to qualified healthcare professionals, such as practice nurses (PNs), offers a potential solution [5–7]. In this paper, the term PN refers to all nurses working in general practice, regardless of qualification level.

International evidence shows that PN-led care can achieve clinical outcomes comparable to physician-led care, with high levels of patient satisfaction [8–11].

Internationally, substantial cross-country differences exist in the definition, training, and existing regulatory frameworks of nursing roles, limiting transferability of the existing evidence, particularly to countries with more restricted delegation models and limited experience with PN-led care [12]. International PN roles range from highly autonomous advanced practice models to predominantly physician-delegated models [12,13].

In Anglo-Saxon countries and some Scandinavian countries, advanced practice nurses, nurse practitioners, and practice nurses provide a higher degree of autonomy, including independent PNLC and, in some countries, prescribing authority [12,13]. In other countries, including Germany, Italy, Belgium, Luxembourg, the Czech Republic, and Austria, legal frameworks further restrict PNs’ roles by prohibiting prescribing, independent consultations, or ordering diagnostics [12].

In Germany, task shifting has gradually expanded through the introduction of specially trained nurses in general practice, such as VERAHs (health care assistants in general practice) and NäPAs (non-physician practice assistants) [14]. In comparison to PNs in German general practice, those practitioners are allowed to perform delegated tasks such as home visits or vaccinations under GP supervision [14]. Additionally, in recent years nursing degree master programs were introduced, reflecting adaption of care models [14,15]. However, the scope of PNs care in Germany still mainly includes monitoring vital signs, assisting with examinations and administrative tasks (Box 1) [14].

**Box 1.**
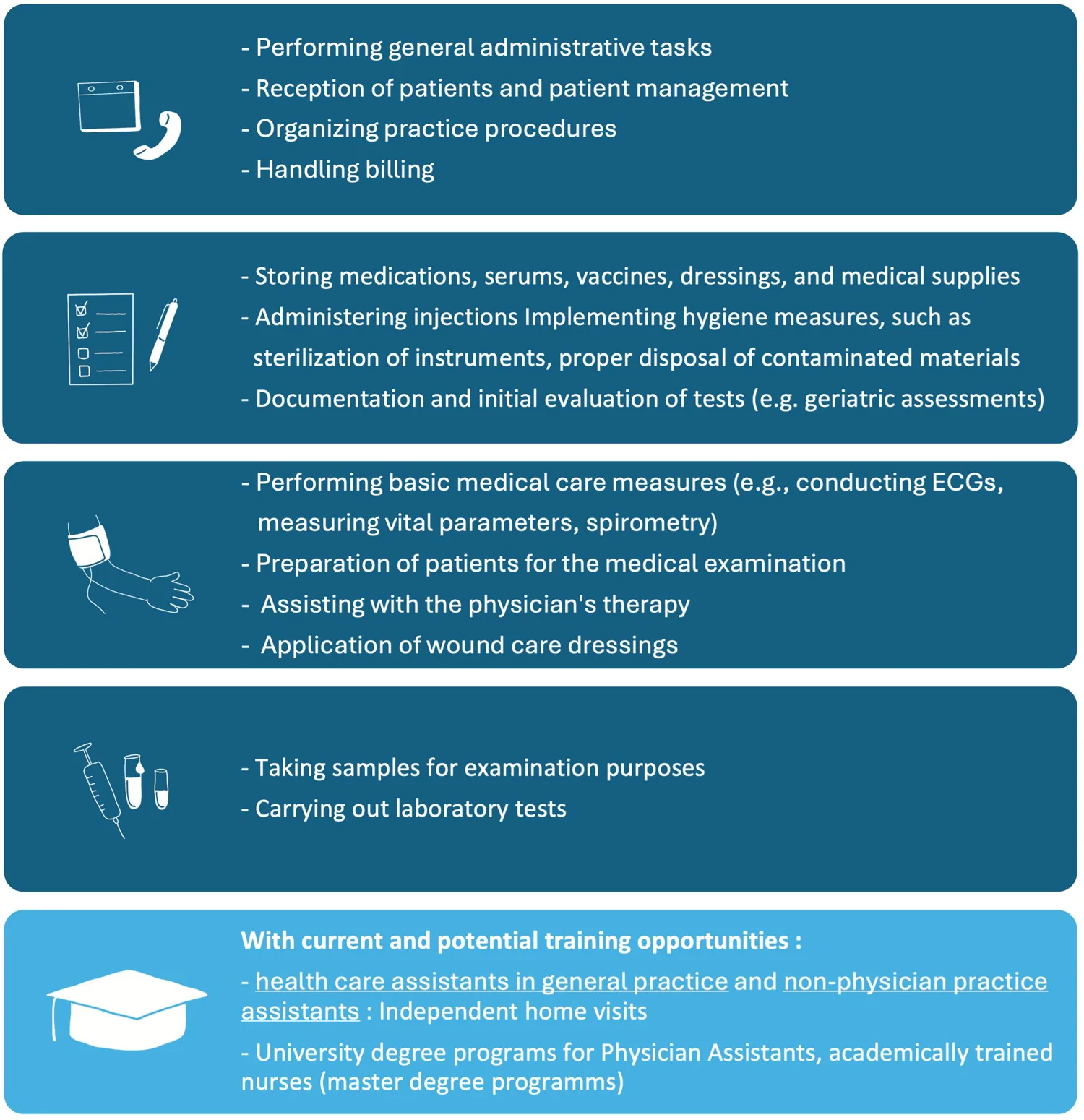
Overview of usual tasks performed by PNs in German GP practice.

Most evidence regarding patient acceptability and outcomes of PN-led care, especially focused on PN-led consultations (PNLC) stems from countries with expanded PN scope of practice such as England, Netherlands or Denmark [11,16–21]. Studies from healthcare systems without established PNLC remain limited [5,22–26].

The structural differences in healthcare systems are particularly relevant for patient-reported outcomes such as acceptance of PNLC, as they shape patients’ familiarity with PNLC and perceptions of professional legitimacy [17,20,22]. Rare available quantitative studies in settings without implemented PNLC suggest generally high acceptance of PNLC, especially for follow-up consultations and minor illnesses, but provide limited insight into which specific consultation conditions patients consider appropriate [5,23,24,26].

Furthermore, evidence regarding whether acceptance differs according to patient characteristics, such as place of residence or chronic disease status, remains limited [26]. Understanding such differences may be particularly relevant for planning future care models in rural areas and for patients with increased healthcare needs.

### Objectives

This study examines general populations’ attitudes towards PNLC, focusing on condition-specific preferences, associated practice-related and individual characteristics, including place of residency and chronic disease status.

## Methods

### Study design

We conducted a cross-sectional survey of 1,532 adults from the Health-Related Beliefs and Health Care Experiences (HeReCa) study in Germany [27].

### Recruitment and selection of study subjects

Participants from the HeReCa panel were recruited between November 2019 and December 2020 across five German federal states (Saxony-Anhalt, Berlin, Schleswig-Holstein, North Rhine-Westphalia, Baden-Württemberg). From randomly chosen 12 to 15 municipalities per state, a total of 50,045 individuals (10,000 per state) aged over 18 years were invited *via* municipal registers and received a web link to join the panel. Recruitment targeted regional representativeness based on the population’s urbanity and age structure at the level of the selected federal states.

Those who did not respond to the initial invitation received one postal reminder, leading to 3,346 enrolled participants. By July 2023, 3,066 people were active in the panel and were invited to participate in our survey.

### Data collection

We conducted our Lime Survey-based survey between July and September 2023, with two email reminders.

### Study outcomes

The primary outcome was participants’ willingness to accept PNLC without GP contact.

Secondary outcomes included participants’ trust in, satisfaction with, and perceived competence of GPs and PNs, assessed using Likert scales (Online Appendix Table A1).

**Table 1.** Respondents socio-demographic characteristics, health characteristics and GP practice-related characteristics (*N* = 1,475).

Variable	n (%)
Gender	
Non-female	608 (43.0%)
Female	805 (57.0%)
Age	
Mean (years)	56.4
23-33 years	115 (7.8%)
34-43 years	180 (12.2%)
44-53 years	242 (16.4%)
54-63 years	431 (29.2%)
64-73 years	373 (25.3%)
74–83 years	134 (9.1%)
Residential federal state	
Berlin	280 (19.0%)
Saxony – Anhalt	301 (20.4%)
North Rhine-Westphalia	253 (17.2%)
Schleswig-Holstein	326 (22.1%)
Baden-Württemberg	315 (21.4%)
Population density	
0–5,000 Inhabitants	194 (13.2%)
5,001–100,000 Inhabitants	775 (52.5%)
≥ 100,000 Inhabitants	506 (34.3%)
Nationality	
Other than German	30 (2.1%)
German	1,381 (97.9%)
Native language	
Other than German	44 (3.1%)
German	1,365 (96.9%)
Marital status	
Married	932 (66.3%)
Single/Divorced/Widowed	474 (33.7%)
Educational level	
Before A-levels	475 (34.1%)
A-levels	918 (65.9%)
Living situation	
Not Alone	1,182 (83.7%)
Alone	230 (16.3%)
Net household income per month	
< 3,000 €	555 (43.6%)
≥ 3,000 €	718 (56.4%)
Chronic condition (any)^¹^	
At least one	961 (65.4%)
None	509 (34.6%)
Arterial hypertension^¹^	529 (36.6%)
Bronchial asthma^¹^	167 (11.6%)
Diabetes mellitus^¹^	124 (8.7%)
Chronic obstructive pulmonary disease (COPD)¹	74 (5.2%)
Thyroid disease^¹^	350 (24.5%)
Chronic back pain^¹^	311 (21.8%)
Coronary artery disease^¹^	99 (6.9%)
Cardiac insufficiency¹	63 (4.4%)
Stroke^¹^	30 (2.1%)
Heart attack^¹^	37 (2.6%)
Years as patient in GP practice	
≤ 10 years	705 (50.8%)
> 10 years	683 (49.2%)
Commuting time to the GP practice (minutes)	
Mean (SD)	15.1 (16.6)
Min, Max	1, 240
Are there any GP practices within the same or shorter distance of your current GP practice?	
Yes	349 (23.9%)
No	1,114 (76.1%)
Number of GP practice-visits in the last 12 months	
≤ 2 times	767 (52.0%)
> 2 times	708 (48.0%)
Practice Organisation	
Single-handed practice	582 (39.7%)
Group practice	883 (60.3%)

GP – general practitioner; SD – standard deviation.

Number of missing and not applicable values are shown in Online Appendix Table A3.

^1^ Self-reported pre-existing condition.

### Measurements and questionnaire

Additionally, we assessed perceived quality of care in GP practice, practice overload, perceived benefits of PNLC and reasons for rejection. Practice-level characteristics and participant factors (e.g. commuting time, chronic conditions, care duration, consultation frequency) were collected. Sociodemographic data (e.g. sex, age, federal state, population density of residence, nationality, native language, marital status, education, living situation) were obtained during HeReCa enrolment.

**Figure 1.**
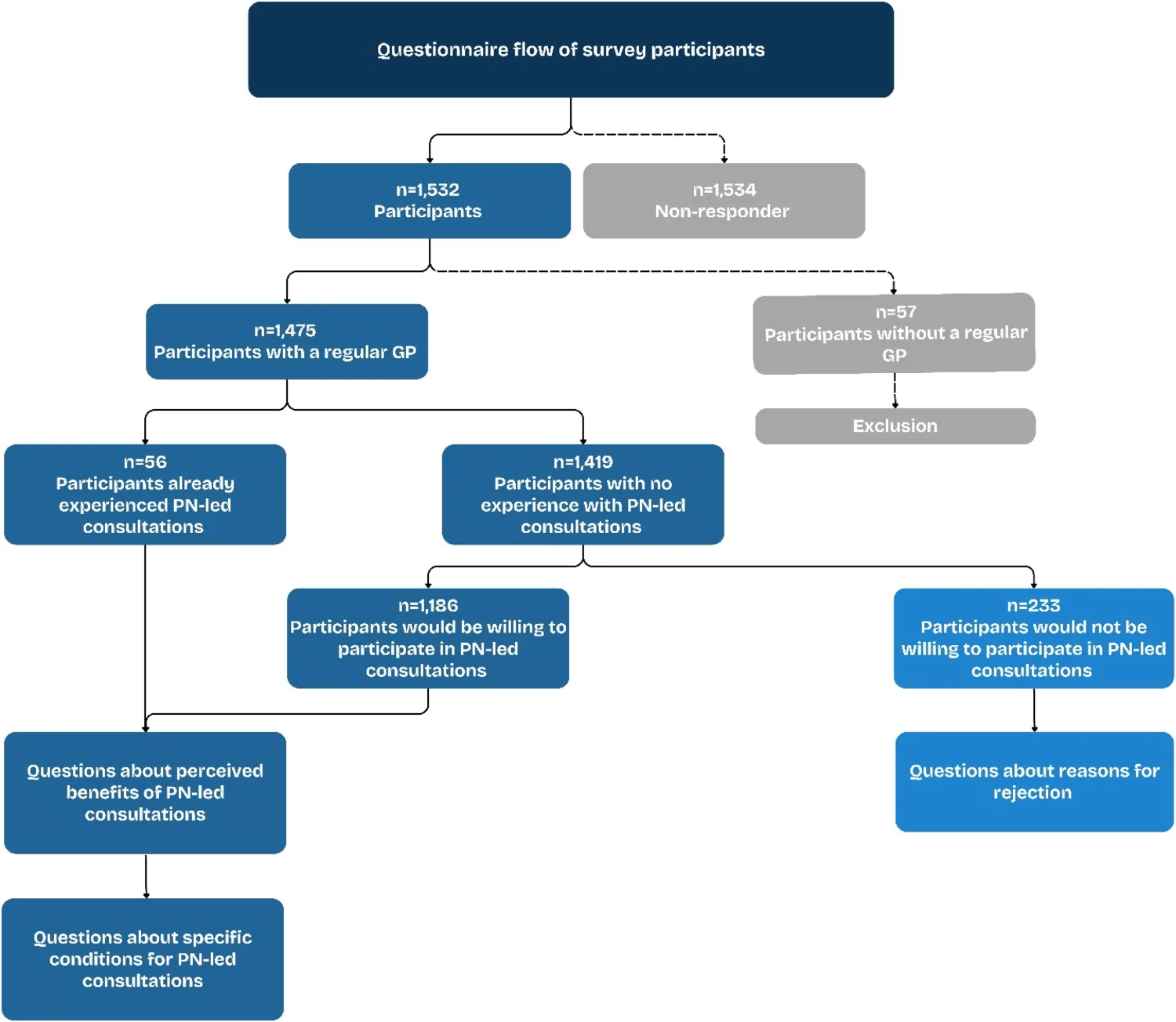
Questionnaire flow of survey participants. Created with Canva.

Only participants with a regular GP were included. The questionnaire obtained conditional routeing guidance (Figure 1).

Since PNLCs are uncommon in Germany, participants were introduced to a hypothetical scenario, describing advanced PN models and PNLC in other countries. Based on prior qualitative work we used a self-developed, pre-tested questionnaire assessing attitudes and condition-specific preferences towards PNLC [25]. In this study, the term ‘conditions’ refers to all issues addressed during patient consultations, including reasons for encounter – e.g. symptoms or preventive care and acute or chronic diagnoses.

We adapted the primary outcome question from Jedro et al.: ‘Would you be willing to attend PNLC without seeing your GP?’, assessing on a 5-point Likert-scale (Online Appendix Table A1) [5]. We categorised those who answered that PNLC ‘already takes place’ as having already experienced PNLC, and all others as ‘no prior experience with PNLC’. Following this approach, willingness was assessed overall and for specific conditions [5].

Items included acute and chronic conditions: superficial injuries, upper respiratory, gastrointestinal and urinary tract infections, new-onset back pain, chronic obstructive pulmonary disease, chronic wounds, bronchial asthma, arterial hypertension, diabetes mellitus, and thyroid diseases (Online Appendix Table A1). The questionnaire is provided as a supplementary file.

### Handling of data

Missing values were excluded. Variables transformation, includes dichotomizations and categorisation, detailed in Online Appendix Table A1.

### Statistical analysis

Descriptive, bivariate and binary logistic regression analyses with multicollinearity tests were performed to assess willingness for PNLC. Multivariable analyses included theoretically relevant predictors and additional variables based on the research team’s experience (i.e. travel time to practice, availability of closer practices) [5,16,19,23,25]. Variables with skewed distributions or strong collinearity were excluded; this affected the variables travel time (correlated with population density), employment status (collinear with education) and income (collinear with education).

Federal state and population density were added separately to the baseline model in sensitivity analyses. Variance Inflation Factor and tolerance values indicated no multicollinearity. Analyses were based on complete cases. Reported Odds Ratios are adjusted. We used IBM SPSS Statistics 25.0 and R Version 4.5.2 (Online Appendix; Software and Packages). This study is reported in accordance with STROBE guidelines (Online Appendix; STROBE statement).

## Results

Of the 3,066 individuals contacted *via* the HeReCa online panel, 1,532 responded to our survey (response proportion: 49.97%), of those 1,475 reported having a GP and were included in the analysis (48.11%; Figure 1).

### Participants’ characteristics

Respondent’s age ranged from 23 to 83 years (mean: 56.4 years); the majority were female (57.0%; Table 1). Most participants (60.3%) attended group practices, and 49.2% had been patients of their GP practice for over 10 years. From all participants, the majority (65.4%) had at least one chronic condition (Table 1).

### Attitudes towards PNLC

#### Prior experience with PNLC

**Figure 2.**
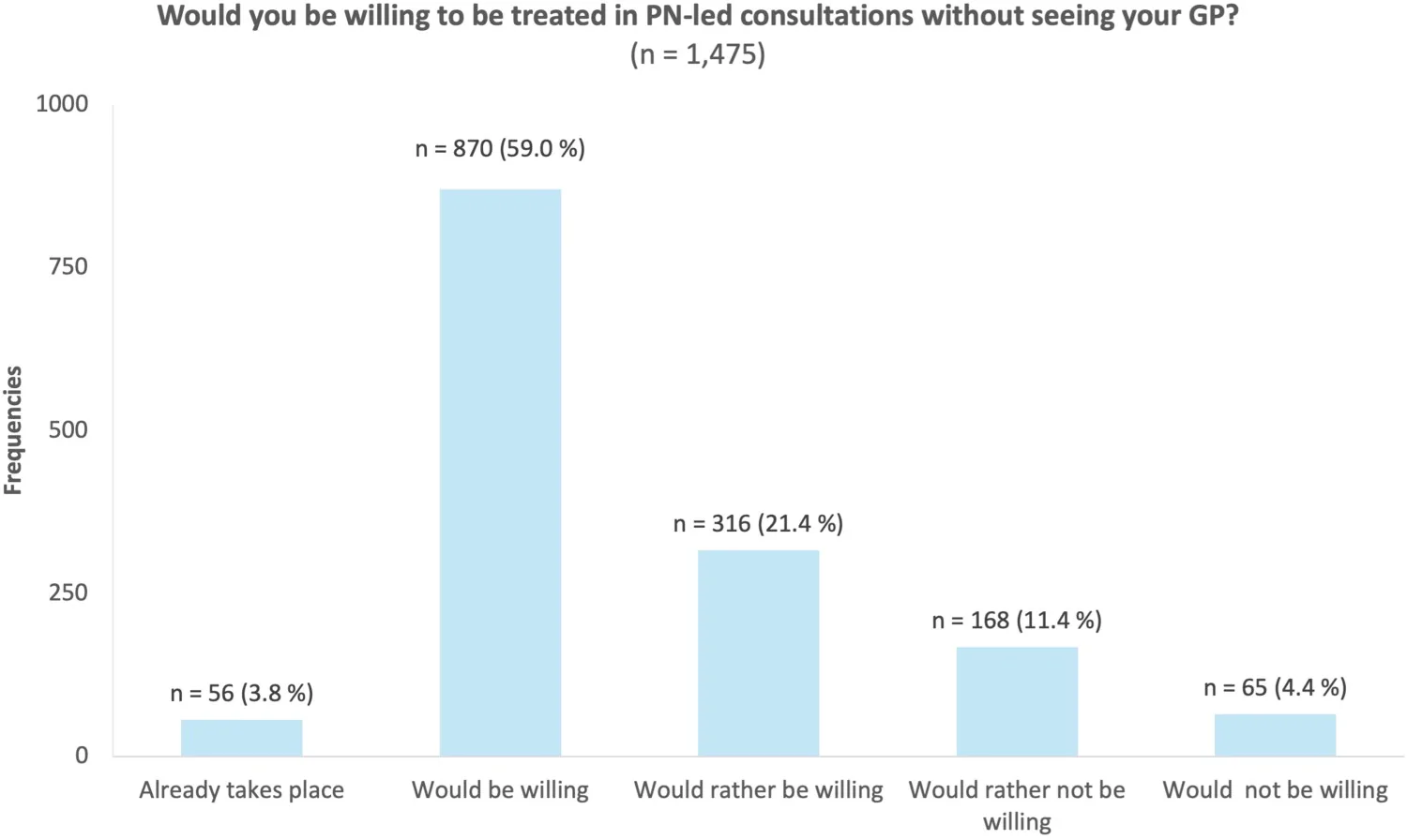
Attitudes towards PN-led patient consultations (*n* = 1,475) This Figure shows willingness to PN-led consultations without GP presence. We categorised participants who answered that PNLC ‘already takes place’ as having already experienced PNLC, and all others as ‘no prior experience with PNLC’.

**Figure 3.**
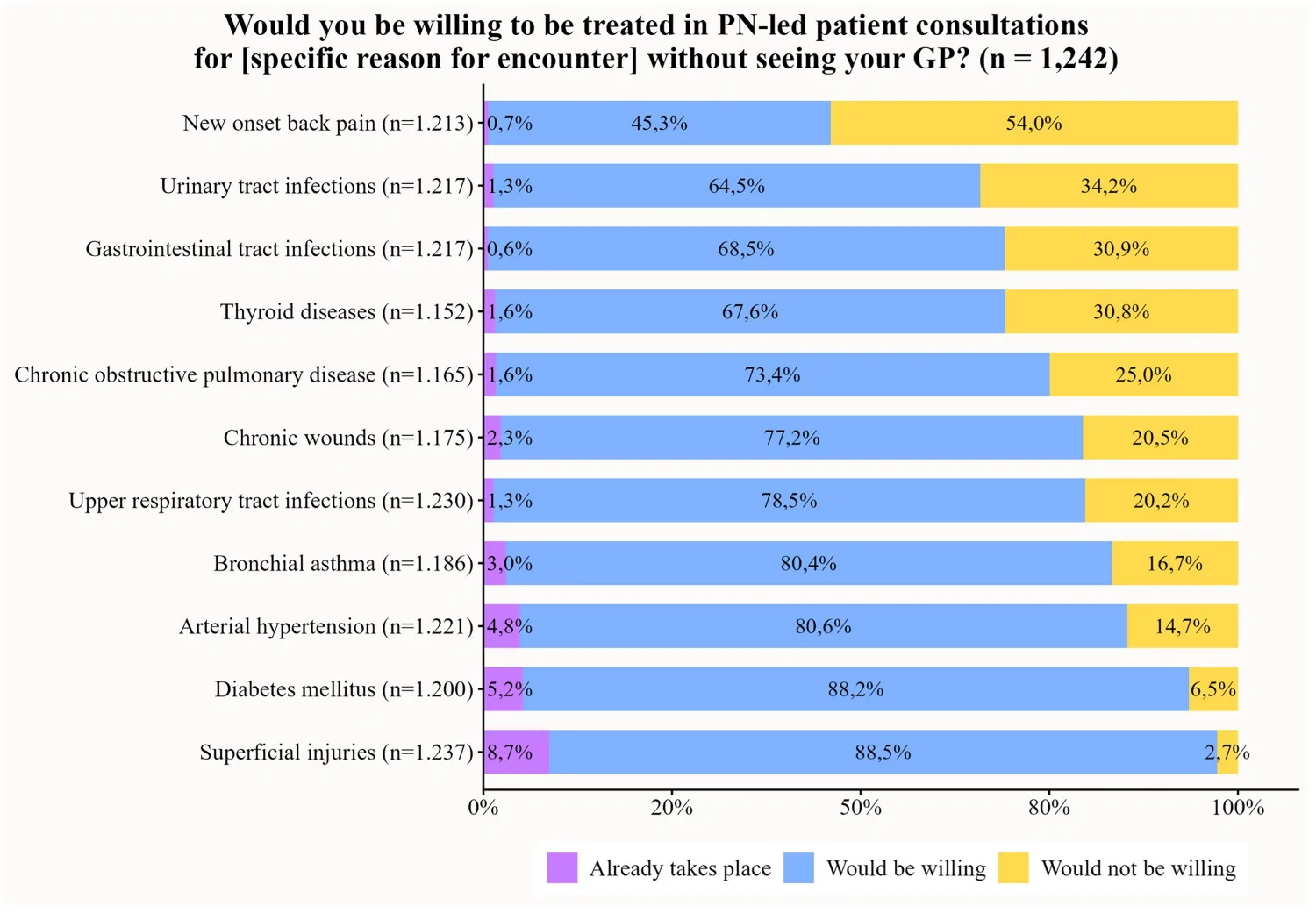
Experiences and attitudes towards PN-led patient consultations for specific reasons for encounter (*n* = 1,242).

Prior experience with PNLC was reported by 3.8% of participants (*n* = 56; Figure 2). Of those, 55.4% were non-female, with a mean age of 59.7 years (Table A2, Online Appendix). Eighty percent reported at least one chronic condition. Nearly half (49.1%) attended group practices and 63.6% had no closer alternative GP practice available. Participants reported high agreement regarding feeling well cared for and trusting the PN’s skills (Table A2, Online Appendix). Participants mostly experienced PNLC for superficial injuries (8.7%), diabetes mellitus (5.2%), and arterial hypertension (4.8%; Figure 3).

#### No prior experience with PNLC

Among those without prior PNLC experience (*n* = 1,419), 42.5% were non-female, 64.8% had at least one chronic condition and 76.6% had no closer practices available. In this group 83.6% would be willing to attend PNLC (Figure 2; Table A2, Online Appendix). When asked about expected benefits of PNLC participation, participants agreed that PNLC would allow more time for their consultations (69.0%), facilitate timely appointment scheduling (68.3%) and improved understanding of their health issues (52.1%; Online Appendix Figure A1).

Acceptance of PNLC for specific conditions was highest for superficial injuries (88.5%), diabetes mellitus (88.2%), arterial hypertension (80.6%), bronchial asthma (80.4%) and upper respiratory tract infections (78.5%; Figure 3).

### Reasons for rejection of PNLC

Reasons for PNLC refusal included agreement that consultations are GP-only tasks (80.3%), a strong preference for consult only with GP (70.9%), and a general rejection of PNLC (42.3%; Online Appendix Figure A2).

### Logistic regression analysis

#### Relevant predictors of PNLC acceptance

**Figure 4.**
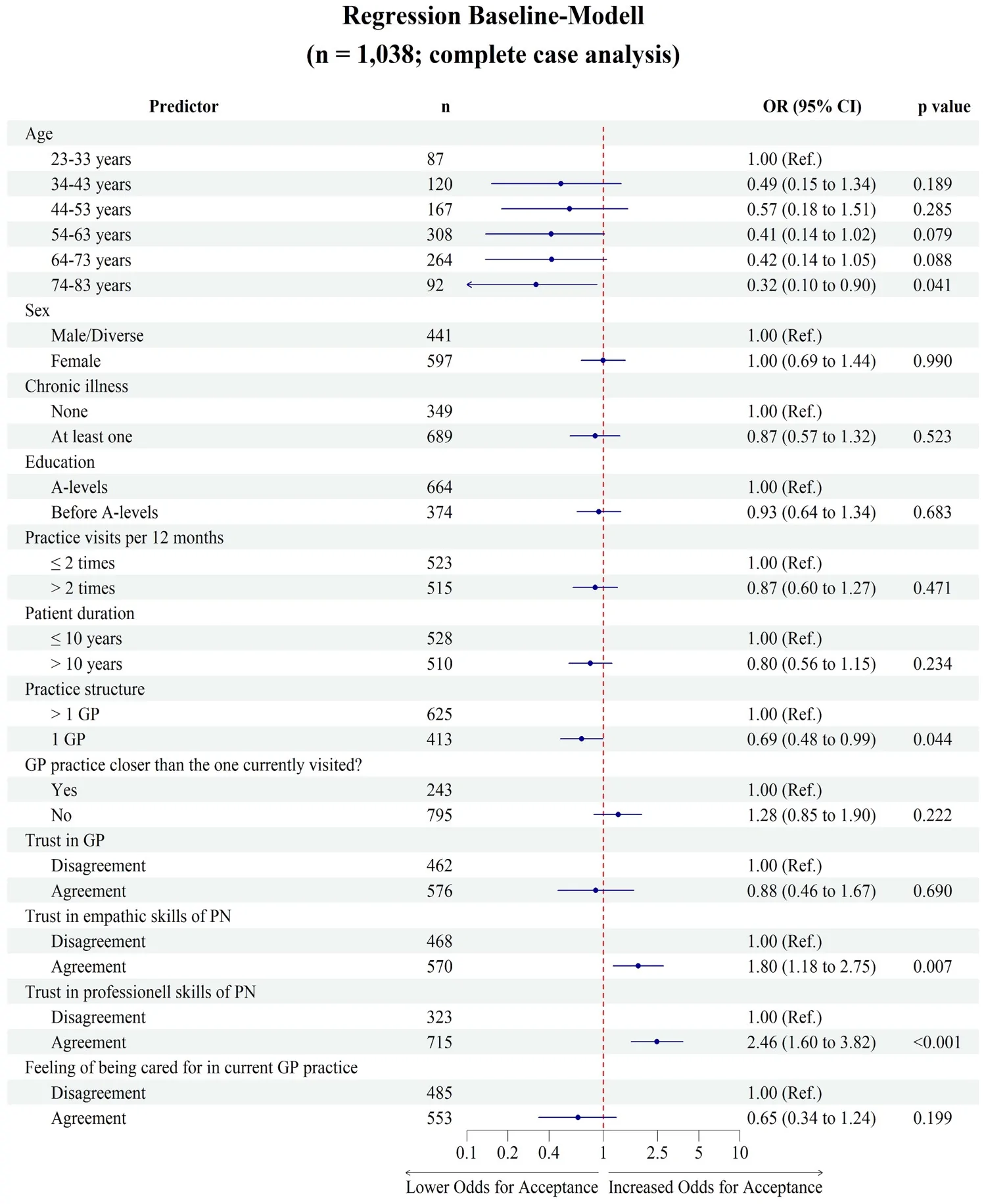
Logistic regression: Baseline Model (complete case analysis *n* = 1,038).

Multivariable analysis confirmed that trust in PNs skills (OR 2.46, 95% CI [1.60;3.82]) and communication (OR 1.80, 95% CI [1.18;2.75]) increased openness to PNLC (Figure 4). Openness was highest among the youngest participants (22–33 years); and lower and comparable across older age groups (OR 0.32; 95% CI [0.10;0.90] for ages 74–83 compared to youngest). Attendance of SHP (OR 0.69; 95% CI [0.48;0.99]; Ref: group practice; Figure 4) was associated with lower PNLC acceptance.

Federal state and population density showed no relevant effects in our sensitivity models (Online Appendix Figures A3 and A4). Associations for the trust variables remained stable across all models: trust in PNs’ communication skills (Online Appendix Figure A3: OR = 1.79, 95% CI [1.17–2.74]; Online Appendix Figure A4: OR = 1.78, 95% CI [1.17–2.73]) and trust in PNs’ professional skills (Online Appendix Figure A3: OR = 2.43, 95% CI [1.57–3.79]; Online Appendix Figure A4: OR = 2.54, 95% CI [1.64–3.95]).

Other variables showed no relevant association with PNLC acceptance in our sample.

Sensitivity analyses with federal state and population density provided similar results and did not indicate regional differences (Online Appendix Figures A3 and A4).

## Discussion

### Main findings

Although PNLC is not permitted under current German delegation regulations, it occurs in practice, suggesting interest and potential need within GP teams to maintain care despite workforce shortages [28]. Participants with prior PNLC experience showed a higher proportion of chronically ill persons and were less likely to have access to nearby GP practices compared with those without prior PNLC experience. Majority reported positive attitudes towards their GP practice and PNs.

PNLC acceptance was high among participants, particularly for superficial wounds, diabetes, hypertension, asthma and upper respiratory tract infections. Trust in PNs communication and professional skills increased PNLC acceptance, while participants over 73 years and those attending SHP were more reluctant. Regional factors and chronic disease status showed no independent association.

### Comparison with existing literature

Existing studies on patient attitudes towards the specific topic of PNLC remain limited and mostly stem from post-implementation or pilot settings [16–21]. Pre-implementation studies are scarce [5,22–26]. Most prior research is qualitative [17,18,21,22,25]; our study adds quantitative data from the general population in PNLC naive country. As most previous studies were conducted in post-implementation settings, comparison with our findings is limited. Analyses were restricted to respondents with a GP. As GPs in Germany provide care for the vast majority of residents, our sample is comparable to a GP’s patient sample.

Consistent with existing literature, younger age was associated with higher PNLC acceptance [5,16,19,23,26]. Trust in PNs’ professional and communication skills was the strongest predictor, confirming the importance of interpersonal aspects [20,21]. Diverging from earlier finding, trust in GPs showed no association with PNLC acceptance [18,21]. Sex showed no effect, whereas earlier studies were inconsistent, showing higher acceptance among men (Germany, Denmark) or women (Scotland) [5,16,19].

Our sample covered structurally heterogeneous regions across Germany [29–31]. Despite variation in practice structure, urbanity and GP density, multivariate analyses showed no independent effect of federal state or population density on PNLC acceptance. This contradicts another German study reporting lower acceptance of expanded PN practice in eastern Germany and Berlin [26]. However, as our study did not cover all 16 German federal states, we found no east-west differences. Likewise, acceptance was not higher in low- than high-density regions, contradicting the hypothesis that rural populations may be more receptive than urban populations [22].

Practice structure, emerged as an associated factor for PNLC willingness: participants from group practices were more open than those from SHP, possibly reflecting greater familiarity with multiple providers fostering trust in competence over personal continuity [32]. This contradicts Danish findings reporting higher acceptance in SHP settings [16]. Prior findings showed: PN experience reduces preference for GP-only consultation, highlighting the role of established PN relationships and patient autonomy [17,19].

Compared with official data, group practices were overrepresented in our sample (60.7% in our sample vs. 32.5% in official data), likely because participant counted all physicians involved in their care, including residents in training leading to an overestimation of group practices [30].

Overall, these findings suggests that meso-level organisational factors appear more relevant than regional characteristics, consistent with prior findings., which observed practice-level spatial effects (ICC = 12.7%) [23].

Finally, our results challenge assumptions that PNLC is accepted only for less complex issues [17,19,20,23]. Participants in our study were willing to consult PNs for 10 of 11 conditions, with highest acceptance for superficial wounds, diabetes, and hypertension, and lowest for new-onset back pain. PNLC acceptance for acute conditions (e.g. bronchial, gastrointestinal, and urinary infections) contrasting with earlier studies that reported more limited acceptance [23]. Earlier studies may have obscured condition-specific effects by aggregating conditions [23,33].

Compared with Jedro et al. who surveyed the nationwide general population without controlling for GP care, we observed higher PNLC acceptance and prior PNLC experience (3.5% vs. 1.5%) [5]. Both studies showed higher acceptance among younger participants, but we identified practice structure as an associated factor, while sex, nationality, and region showed no relevant effect [5].

Kuschick et al. recruited patients from general practices in three federal states, assessing PNLC openness for current and broad hypothetical subgroups [23]. We extended this by examining acceptance across more conditions and five federal states, achieving wider spatial coverage, but showing no relevant regional impact [23]. Overall, both studies indicate higher PNLC acceptance for follow-up consultations (e.g. chronic cases) [23].

Jäkel et al. reported acceptance 52.8% of extended PN roles in a large nationwide German sample (*n* = 6,733), consisting with our findings of willingness towards PNLC [26].

### Strengths

This is the first German cross-sectional quantitative study assessing public attitudes towards PNLC for a broad range of specific conditions. Previous studies in this field examined openness for overall chronic or acute conditions, general uncomplicated problems, or specific consultations (e.g. follow-ups, routine checks), but not across a variety of specific conditions [5,16,17,20,23,26]. A comprehensive set of variables, including high-resolution regional data, allowed a multifactorial explanation of influencing factors. The high response rate (49.97%) shows strong participant engagement. Furthermore, the comparatively large sample enables a robust basis for statistical analyses and enhances reliability.

Fifthly, the questionnaire builds on prior qualitative studies and was adapted from established instruments [5,25,34]. Lastly, structured filtering enabled focused analysis of regular GP patients.

### Limitations

Despite the high response rate, non-responder bias cannot be excluded. Participants’ awareness of the study aims may have led some participants who were less open to PNLC to decline, potentially overestimating acceptance. Social desirability may have inflated responses. Heterogeneous prior PNLC experiences and differences in international PN roles limit generalisability. Practice-level clustering seems unlikely but cannot be excluded. All data were self-reported, so recall bias cannot be excluded. Older and socioeconomically disadvantaged groups were underrepresented. The cross-sectional design excludes causal inferences.

### Implications for clinicians and policymakers

Clinicians and policymakers should note that large parts of the general adult population are open to PNLC overall and for specific conditions. Informal PNLC experience despite restrictive legal frameworks suggests practical innovations are outpacing legal developments in Germany. Legal frameworks should be adapted to support evolving delegation models.

PN-led follow-up consultations for chronic (e.g. hypertension, diabetes) and acute conditions (e.g. seasonal infections) appear acceptable. Regions with experience in team-based care, such in group practices, and younger populations and may be particularly receptive, facilitating PNLC implementation.

### Suggestions for further research

Future research should examine GPs’ and PNs’ perspectives to guide PNLC implementation. Pilot projects should focus on PN-led care for chronic conditions with highest acceptance (e.g. diabetes, hypertension, asthma) and evaluate supervision models ensuring patient safety while improving access and reducing workload.

Future research should assess the non-inferiority of PNLC compared with GP-led care and explore appropriate compensation models, including integration into billing structures, to support and incentivise advanced PN roles.

## Conclusion

Openness to PNLC is high among the general population, and some German practices have already adopted PNLC despite limited legal frameworks. The results underscore the need for policy action, including expanded regulatory frameworks, supervision requirements and reimbursement options. Initial implementation through pilot projects, supervision models, and further PN training should focus on well-accepted consultation reasons, such as superficial injuries, diabetes, hypertension, asthma, and respiratory infections.

Younger participants tend to be more open to PNLC and could be a target group for initial implementation. As patients’ trust in PNs is strongly associated with openness, further studies should account for this factor. As group practices attendees are more open to PNLC, group practices may represent a suitable starting point for implementation.

## Supplementary Material

Supplemental Material

## Data Availability

Materials and data can be reasonable obtained by contacting the first authors. The datasets generated and analysed during the current study are available from the corresponding author on reasonable request. The study is registered in the German Clinical Trial Register (DRKS, Registration No: DRKS00032386; https://www.drks.de/search/de/trial/DRKS000323).
