# Supplementary material for: Are practice nurse-led patient consultations acceptable for the general population in Germany? Results from a population-based survey
Source: Eur J Gen Pract. 2026 Aug 27;32(1):2716980. doi: 10.1080/13814788.2026.2716980 (PMC13523119; doi:10.1080/13814788.2026.2716980)
Supplement: Supplementary file 1 [file IGEN_A_2716980_SM7054.docx]

**Appendix**

**Table A1: Variable Categorization**

| **Variable** | **Original Scale** | **Transformation** | **Categories/Cut offs** |
| --- | --- | --- | --- |
| **Willingness to accept PNLC**  **(Primary outcome variable)** | 5- Point Likert Scale  1 = Already takes place;  2 = Would be willing;  3 = Would rather be willing;  4 = Would rather not be willing;  5 = Would not be willing | Dichotomized | Would be willing (2-3) / (4-5) Would not be willing |
| **PNLC experience** | As above | Dichotomized | Already takes place = experienced (1)/ Not taking place = Without previous experience (2-5) |
| **Willingness to accept PNLC for specific conditions** | 7- Point Likert Scale  1 = Already takes place;  2 = Would be willing;  3 = Would rather be willing;  4 = Neutral;  5 = Would rather not be willing; 6 = Would not be willing;  7 = Not sure | Categorized | Already takes place (1) / Would be willing (2-4) /Would not be willing (5-6) |
| **Reasons for rejection of PNLC** | 6- Point Likert Scale  1 = Strong agreement;  2= Partial agreement;  3= Neutral;  4= Patrial disagreement;  5 = Strong disagreement;  6 = Not sure | Dichotomized | Agreement (1-2)  Disagreement (3-5) |
| **Perceived benefits of PNLC** | 6- Point Likert Scale  1 = Strong agreement;  2= Partial agreement;  3= Neutral;  4= Patrial disagreement;  5 = Strong disagreement;  6 = Not sure | Dichotomized | Agreement (1-2)  Disagreement (3-5) |
| **Age** | Metric Scale | 10-year intervals | 22-33/ 34-43 / up to 74-83 years |
| **Educational level** | Multiple degrees | Dichotomized | Below A-levels / A-level or above |
| **Chronic condition** | Count of self-reported diseases | Dichotomized | 0 = None / ≥1 At least one |
| **Number of GPs in practice** | 1= 1 GP  2 = > 1 GP | Renamed | 1 = Single-handed practice  2 = Group practice |
| **Trust in PN’s professional skills** | 6- Point Likert Scale  1 = Strong agreement;  2= Partial agreement;  3= Neutral;  4= Patrial disagreement;  5 = Strong disagreement;  6 = Not sure | Dichotomized | Agreement = Higher Trust (1-2)  Disagreement = Lower Trust (3-5) |
| **Trust in PN’s empathy** | 6- Point Likert Scale  1 = Strong agreement;  2= Partial agreement;  3= Neutral;  4= Patrial disagreement;  5 = Strong disagreement;  6 = Not sure | Dichotomized | Agreement = Higher Trust (1-2)  Disagreement = Lower Trust (3-5) |
| **Sense of being well cared for at the current GP practice** | 6- Point Likert Scale  1 = Strong agreement;  2= Partial agreement;  3= Neutral;  4= Patrial disagreement;  5 = Strong disagreement;  6 = Not sure | Dichotomized | Agreement (1)  Disagreement (2-5) |
| **Trust in GP** | 6- Point Likert Scale  1 = Strong agreement;  2= Partial agreement;  3= Neutral;  4= Patrial disagreement;  5 = Strong disagreement;  6 = Not sure | Dichotomized | Agreement= Higher Trust (1)  Disagreement= Lower Trust (2-5) |

**Table A2: Comparison between participants with experience of PN-led consultations and without experience of PN-led consultations**

|  | **no experience vs. experience regarding PN-led patient consultations*^1^*** | | |
| --- | --- | --- | --- |
| **Characteristic** | **Prior experience with PNLC** N = 56*^2^* | **No prior experience with PNLC** N = 1,419*^2^* | **p-value***^3^* |
| **Gender** |  |  | 0.057 |
| Non-female | 31 (55.4%) | 577 (42.5%) |  |
| Female | 25 (44.6%) | 780 (57.5%) |  |
| (Missing or not applicable) | 0 | 62 |  |
| **Age** |  |  | 0.306 |
| Mean (years) | 59.7 | 56.4 |  |
| 23-33 years | 2 (3.6%) | 113 (8.0%) |  |
| 34-43 years | 4 (7.1%) | 176 (12.4%) |  |
| 44-53 years | 11 (19.6%) | 231 (16.3%) |  |
| 54-63 years | 13 (23.2%) | 418 (29.5%) |  |
| 64-73 years | 19 (33.9%) | 354 (24.9%) |  |
| 74-83 years | 7 (12.5%) | 127 (8.9%) |  |
| **Residential federal state** |  |  | 0.125 |
| Berlin | 14 (25.0%) | 266 (18.7%) |  |
| Saxony – Anhalt | 10 (17.9%) | 291 (20.5%) |  |
| North Rhine-Westphalia | 3 (5.4%) | 250 (17.6%) |  |
| Schleswig-Holstein | 16 (28.6%) | 310 (21.8%) |  |
| Baden-Württemberg | 13 (23.2%) | 302 (21.3%) |  |
| **Population density** |  |  | 0.363 |
| 0 – 5,000 Inhabitants | 10 (17.9%) | 184 (13.0%) |  |
| 5,001 – 100,000 Inhabitants | 31 (55.4%) | 744 (52.4%) |  |
| ≥ 100,000 Inhabitants | 15 (26.8%) | 491 (34.6%) |  |
| **Nationality** |  |  | >0.999 |
| Other than German | 1 (1.8%) | 29 (2.1%) |  |
| German | 54 (98.2%) | 1,327 (97.9%) |  |
| (Missing or not applicable) | 1 | 63 |  |
| **Native language** |  |  | >0.999 |
| Other than German | 1 (1.8%) | 43 (3.2%) |  |
| German | 55 (98.2%) | 1,310 (96.8%) |  |
| (Missing or not applicable) | 0 | 66 |  |
| **Marital status** |  |  | 0.875 |
| Married | 37 (67.3%) | 895 (66.2%) |  |
| Single/Divorced/Widowed | 18 (32.7%) | 456 (33.8%) |  |
| (Missing or not applicable) | 1 | 68 |  |
| **Educational level** |  |  | 0.864 |
| Before A-levels | 19 (35.2%) | 456 (34.1%) |  |
| A-levels | 35 (64.8%) | 883 (65.9%) |  |
| (Missing or not applicable) | 2 | 80 |  |
| **Living situation** |  |  | 0.488 |
| Not Alone | 45 (80.4%) | 1,137 (83.8%) |  |
| Alone | 11 (19.6%) | 219 (16.2%) |  |
| (Missing or not applicable) | 0 | 63 |  |
| **Net household income per month** |  |  | 0.051 |
| < 3,000 € | 31 (56.4%) | 524 (43.0%) |  |
| ≥ 3,000 € | 24 (43.6%) | 694 (57.0%) |  |
| (Missing or not applicable) | 1 | 201 |  |
| **Chronic condition (any)***^5^* |  |  | 0.016 |
| At least one | 45 (80.4%) | 916 (64.8%) |  |
| None | 11 (19.6%) | 498 (35.2%) |  |
| (Missing or not applicable) | 0 | 5 |  |
| **Arterial hypertension***^5^* | 26 (46.4%) | 503 (36.2%) | 0.121 |
| (Missing or not applicable) | 0 | 31 |  |
| **Bronchial asthma***^5^* | 10 (17.9%) | 157 (11.3%) | 0.135 |
| (Missing or not applicable) | 0 | 34 |  |
| **Diabetes mellitus***^5^* | 14 (25.0%) | 110 (8.0%) | <0.001 |
| (Missing or not applicable) | 0 | 52 |  |
| **Chronic obstructive pulmonary disease (COPD)***^5^* | 6 (10.7%) | 68 (5.0%) | 0.066 |
| (Missing or not applicable) | 0 | 51 |  |
| **Thyroid disease***^5^* | 18 (32.1%) | 332 (24.1%) | 0.172 |
| (Missing or not applicable) | 0 | 44 |  |
| **Chronic back pain***^5^* | 17 (30.9%) | 294 (21.4%) | 0.094 |
| (Missing or not applicable) | 1 | 46 |  |
| **Coronary artery disease***^5^* | 6 (10.7%) | 93 (6.8%) | 0.274 |
| (Missing or not applicable) | 0 | 42 |  |
| **Cardiac insufficiency***^5^* | 3 (5.5%) | 60 (4.4%) | 0.733 |
| (Missing or not applicable) | 1 | 56 |  |
| **Stroke***^5^* | 5 (9.1%) | 25 (1.8%) | 0.005 |
| (Missing or not applicable) | 1 | 54 |  |
| **Heart attack***^5^* | 6 (11.1%) | 31 (2.3%) | 0.002 |
| (Missing or not applicable) | 2 | 56 |  |
| **Years as patient in GP practice** |  |  | 0.174 |
| ≤ 10 years | 23 (41.8%) | 682 (51.2%) |  |
| > 10 years | 32 (58.2%) | 651 (48.8%) |  |
| (Missing or not applicable) | 1 | 86 |  |
| **Commuting time to the GP practice (minutes)** |  |  | 0.085 |
| Mean (SD) | 17.3 (13.2) | 15.0 (16.7) |  |
| Min, Max | 3, 60 | 1, 240 |  |
| (Missing or not applicable) | 0 | 9 |  |
| **Are there any GP practices within the same or shorter distance of your current GP practice?** |  |  | 0.027 |
| Yes | 20 (36.4%) | 329 (23.4%) |  |
| No | 35 (63.6%) | 1,079 (76.6%) |  |
| (Missing or not applicable) | 1 | 11 |  |
| **Number of GP practice-visits in the last 12 months** |  |  | 0.163 |
| ≤ 2 times | 24 (42.9%) | 743 (52.4%) |  |
| > 2 times | 32 (57.1%) | 676 (47.6%) |  |
| **Practice Organization** |  |  | 0.084 |
| Single-handed practice | 28 (50.9%) | 554 (39.3%) |  |
| Group practice | 27 (49.1%) | 856 (60.7%) |  |
| (Missing or not applicable) | 1 | 9 |  |
| **Ability of GP practice’s PNs to discuss medical issues empathically***^6^* |  |  | <0.001 |
| Agreement | 47 (88.7%) | 658 (54.7%) |  |
| Disagreement | 6 (11.3%) | 545 (45.3%) |  |
| (Missing or not applicable) | 3 | 216 |  |
| **Trust in the professional skills of the GP practice’s PNs***^6^* |  |  | <0.001 |
| Agreement | 51 (92.7%) | 888 (68.6%) |  |
| Disagreement | 4 (7.3%) | 407 (31.4%) |  |
| (Missing or not applicable) | 1 | 124 |  |
| **Trust in GP*^7^*** |  |  | 0.053 |
| Agreement | 37 (66.1%) | 748 (52.9%) |  |
| Disagreement | 19 (33.9%) | 666 (47.1%) |  |
| (Missing or not applicable) | 0 | 5 |  |
| **Sense of being well cared for at the current GP practice***^7^* |  |  | 0.009 |
| Agreement | 39 (69.6%) | 728 (51.7%) |  |
| Disagreement | 17 (30.4%) | 679 (48.3%) |  |
| (Missing or not applicable) | 0 | 12 |  |

**Symbols and Abbreviations: n – absolute number of cases; PN – practice nurse, GP – general practitioner, PNLC – practice nurse-led consultation;**

*^1^* Would be willing / Would rather be willing (Yes) vs. Would rather not be willing / Would not be willing (No)

*^2^* n (%)

*^3^* Pearson’s Chi-squared test; Fisher’s exact test; NA; Wilcoxon rank sum test

*^4^* Low = 0–5,000 Inhabitants; Medium = 5,001–100,000 Inhabitants; High ≥ 100,000 Inhabitants

*^5^* Self-reported pre-existing condition

*^6^* Agreement includes “Strongly Agree” and “Somewhat Agree”; Disagreement includes “Neither agree nor disagree”, “Somewhat Disagree”, and “Strongly Disagree”

*^7^* Agreement includes “Strongly Agree”; Disagreement includes “Somewhat Agree”, “Neither agree nor disagree”, “Somewhat Disagree”, and “Strongly Disagree”

**Figure A1: Perceived Benefits of PN-Led Consultations**

**Abbreviations: n – absolute number of cases; PN – practice nurse**

**Figure A2: Reasons for Rejection of PN-led Consultation**

**Abbreviations: n – absolute number of cases; PN – practice nurse; GP – general practitioner; PNLC – practice nurse led consultation**

**Figure A3: Logistic regression model B (Baseline Model + Federal State; Complete case analysis n = 1,038)**

**Abbreviations: n – absolute number of cases; OR – odds Ratio; CI – confidence Interval; GP – general practitioner; PN – practice nurse**

**Figure A4: Logistic regression model C (Baseline Model + Population density; Complete case analysis n = 1,038**

**Abbreviations: n – absolute number of cases; OR – odds Ratio; CI – confidence Interval; GP – general practitioner; PN – practice nurse**

**Tab**

**Table A3: Missing or not applicable values for sociodemographic, health and GP practice-related characteristics among all respondents (N =1,475)**

| **Variable** | **n** |
| --- | --- |
| Gender | 62 |
| Age | - |
| Residential federal state | - |
| Population density | - |
| Nationality | 64 |
| Native language | 66 |
| Marital status | 69 |
| Educational level | 82 |
| Living situation | 63 |
| Net household income per month | 202 |
| Chronic condition (any) | 5 |
| Arterial hypertension | 31 |
| Bronchial asthma | 34 |
| Diabetes mellitus | 52 |
| Chronic obstructive pulmonary disease (COPD) | 51 |
| Thyroid disease | 44 |
| Chronic back pain | 47 |
| Coronary artery disease | 42 |
| Cardiac insufficiency | 57 |
| Stroke | 55 |
| Heart attack | 58 |
| Years as patient in GP practice | 87 |
| Duration of travel time to the GP practice (minutes) | 9 |
| Are there any GP practices within the same or shorter distance of your current GP practice? | 12 |
| Number of GP practice-visits in the last 12 months | - |
| Practice Organization | 10 |

Abbreviations: **n – absolute number of cases; GP – general practitioner; PN – practice nurse, SD -** standard deviation

**Software and Packages**

Statistical analyses were conducted using:

IBM SPSS Statistics for Windows (Version 25.0; IBM Corp., 2017) and

R Development Core Team. R: A Language and Environment for Statistical Computing. Version 4.5.2 ed. Vienna, Austria: R Foundation for Statistical Computing; 2025.

The following R packages were used:

  - Arel-Bundock V, Greifer N, Heiss A (2024). “How to Interpret Statistical Models Using marginaleffects for R and Python.” _Journal of Statistical Software_, *111*(9), 1-32. doi:10.18637/jss.v111.i09 <https://doi.org/10.18637/jss.v111.i09>.
  - Dayimu A (2025). _forestploter: Create a Flexible Forest Plot_. doi:10.32614/CRAN.package.forestploter <https://doi.org/10.32614/CRAN.package.forestploter>, R package version 1.1.3, <https://CRAN.R-project.org/package=forestploter>.
  - Fox J, Weisberg S (2019). _An R Companion to Applied Regression_, Third edition. Sage, Thousand Oaks CA. <https://www.john-fox.ca/Companion/>.
  - Fox J, Weisberg S, Price B (2022). _carData: Companion to Applied Regression Data Sets_. doi:10.32614/CRAN.package.carData <https://doi.org/10.32614/CRAN.package.carData>, R package version 3.0-5, <https://CRAN.R-project.org/package=carData>.
  - Grolemund G, Wickham H (2011). “Dates and Times Made Easy with lubridate.” _Journal of Statistical Software_, *40*(3), 1-25. <https://www.jstatsoft.org/v40/i03/>.
  - Hebbali A (2024). _olsrr: Tools for Building OLS Regression Models_. doi:10.32614/CRAN.package.olsrr <https://doi.org/10.32614/CRAN.package.olsrr>, R package version 0.6.1, <https://CRAN.R-project.org/package=olsrr>.
  - Iannone R, Cheng J, Schloerke B, Hughes E, Lauer A, Seo J, Brevoort K, Roy O (2025). _gt: Easily Create Presentation-Ready Display Tables_. doi:10.32614/CRAN.package.gt <https://doi.org/10.32614/CRAN.package.gt>, R package version 1.1.0, <https://CRAN.R-project.org/package=gt>.
  - Larmarange J, Sjoberg D (2025). _broom.helpers: Helpers for Model Coefficients Tibbles_. doi:10.32614/CRAN.package.broom.helpers <https://doi.org/10.32614/CRAN.package.broom.helpers>, R package version 1.22.0, <https://CRAN.R-project.org/package=broom.helpers>.
  - Lele SR, Keim JL, Solymos P (2023). _ResourceSelection: Resource Selection (Probability) Functions for Use-Availability Data_. doi:10.32614/CRAN.package.ResourceSelection <https://doi.org/10.32614/CRAN.package.ResourceSelection>, R package version 0.3-6, <https://CRAN.R-project.org/package=ResourceSelection>.
  - Lüdecke D (2025). _sjPlot: Data Visualization for Statistics in Social Science_. R package version 2.9.0, <https://CRAN.R-project.org/package=sjPlot>.
  - Makowski D, Lüdecke D, Patil I, Thériault R, Ben-Shachar M, Wiernik B (2023). “Automated Results Reporting as a Practical Tool to Improve Reproducibility and Methodological Best Practices Adoption.” _CRAN_. doi:10.32614/CRAN.package.report <https://doi.org/10.32614/CRAN.package.report>, <https://easystats.github.io/report/>.
  - Müller K (2025). _here: A Simpler Way to Find Your Files_. doi:10.32614/CRAN.package.here <https://doi.org/10.32614/CRAN.package.here>, R package version 1.0.2, <https://CRAN.R-project.org/package=here>.
  - Müller K, Wickham H (2025). _tibble: Simple Data Frames_. doi:10.32614/CRAN.package.tibble <https://doi.org/10.32614/CRAN.package.tibble>, R package version 3.3.0, <https://CRAN.R-project.org/package=tibble>.
  - Patil I (2021). “Visualizations with statistical details: The 'ggstatsplot' approach.” _Journal of Open Source Software_, *6*(61), 3167. doi:10.21105/joss.03167 <https://doi.org/10.21105/joss.03167>, <https://doi.org/10.21105/joss.03167>.
  - Pedersen T (2025). _patchwork: The Composer of Plots_. doi:10.32614/CRAN.package.patchwork <https://doi.org/10.32614/CRAN.package.patchwork>, R package version 1.3.2, <https://CRAN.R-project.org/package=patchwork>.
  - R Core Team (2025). _R: A Language and Environment for Statistical Computing_. R Foundation for Statistical Computing, Vienna, Austria. <https://www.R-project.org/>.
  - Robin X, Turck N, Hainard A, Tiberti N, Lisacek F, Sanchez J, Müller M (2011). “pROC: an open-source package for R and S+ to analyze and compare ROC curves.” _BMC Bioinformatics_, *12*, 77.
  - Robinson D, Hayes A, Couch S (2025). _broom: Convert Statistical Objects into Tidy Tibbles_. doi:10.32614/CRAN.package.broom <https://doi.org/10.32614/CRAN.package.broom>, R package version 1.0.10, <https://CRAN.R-project.org/package=broom>.
  - Sjoberg D, Whiting K, Curry M, Lavery J, Larmarange J (2021). “Reproducible Summary Tables with the gtsummary Package.” _The R Journal_, *13*, 570-580. doi:10.32614/RJ-2021-053 <https://doi.org/10.32614/RJ-2021-053>, <https://doi.org/10.32614/RJ-2021-053>.
  - Wickham H (2016). _ggplot2: Elegant Graphics for Data Analysis_. Springer-Verlag New York. ISBN 978-3-319-24277-4, <https://ggplot2.tidyverse.org>.
  - Wickham H (2025). _forcats: Tools for Working with Categorical Variables (Factors)_. doi:10.32614/CRAN.package.forcats <https://doi.org/10.32614/CRAN.package.forcats>, R package version 1.0.1, <https://CRAN.R-project.org/package=forcats>.
  - Wickham H (2025). _stringr: Simple, Consistent Wrappers for Common String Operations_. doi:10.32614/CRAN.package.stringr <https://doi.org/10.32614/CRAN.package.stringr>, R package version 1.5.2, <https://CRAN.R-project.org/package=stringr>.
  - Wickham H, Averick M, Bryan J, Chang W, McGowan LD, François R, Grolemund G, Hayes A, Henry L, Hester J, Kuhn M, Pedersen TL, Miller E, Bache SM, Müller K, Ooms J, Robinson D, Seidel DP, Spinu V, Takahashi K, Vaughan D, Wilke C, Woo K, Yutani H (2019). “Welcome to the tidyverse.” _Journal of Open Source Software_, *4*(43), 1686. doi:10.21105/joss.01686 <https://doi.org/10.21105/joss.01686>.
  - Wickham H, François R, Henry L, Müller K, Vaughan D (2023). _dplyr: A Grammar of Data Manipulation_. doi:10.32614/CRAN.package.dplyr <https://doi.org/10.32614/CRAN.package.dplyr>, R package version 1.1.4, <https://CRAN.R-project.org/package=dplyr>.
  - Wickham H, Henry L (2025). _purrr: Functional Programming Tools_. doi:10.32614/CRAN.package.purrr <https://doi.org/10.32614/CRAN.package.purrr>, R package version 1.1.0, <https://CRAN.R-project.org/package=purrr>.
  - Wickham H, Hester J, Bryan J (2024). _readr: Read Rectangular Text Data_. doi:10.32614/CRAN.package.readr <https://doi.org/10.32614/CRAN.package.readr>, R package version 2.1.5, <https://CRAN.R-project.org/package=readr>.
  - Wickham H, Miller E, Smith D (2025). _haven: Import and Export 'SPSS', 'Stata' and 'SAS' Files_. doi:10.32614/CRAN.package.haven <https://doi.org/10.32614/CRAN.package.haven>, R package version 2.5.5, <https://CRAN.R-project.org/package=haven>.
  - Wickham H, Pedersen T, Seidel D (2025). _scales: Scale Functions for Visualization_. doi:10.32614/CRAN.package.scales <https://doi.org/10.32614/CRAN.package.scales>, R package version 1.4.0, <https://CRAN.R-project.org/package=scales>.
  - Wickham H, Vaughan D, Girlich M (2024). _tidyr: Tidy Messy Data_. doi:10.32614/CRAN.package.tidyr <https://doi.org/10.32614/CRAN.package.tidyr>, R package version 1.3.1, <https://CRAN.R-project.org/package=tidyr>.

**STROBE Statement**

|  | Item No. | Recommendation | Page  No. | Relevant text from manuscript |
| --- | --- | --- | --- | --- |
| Title and abstract | 1 | (a) Indicate the study’s design with a commonly used term in the title or the abstract | 1 | Title: ‘… Results from a Population-Based Survey’ |
|  |  | (b) Provide in the abstract an informative and balanced summary of what was done and what was found | 3-4 | Abstract |
| Introduction | | | |  |
| Background/rationale | 2 | Explain the scientific background and rationale for the investigation being reported | 5-6 | Section: Introduction |
| Objectives | 3 | State specific objectives, including any prespecified hypotheses | 6 | Section: Objectives |
| Methods | | | |  |
| Study design | 4 | Present key elements of study design early in the paper | 7 | Section: Study design |
| Setting | 5 | Describe the setting, locations, and relevant dates, including periods of recruitment, exposure, follow-up, and data collection | 7 | Sections: Recruitment and selection of study subjects, Data collection |
| Participants | 6 | (a) Cohort study—Give the eligibility criteria, and the sources and methods of selection of participants. Describe methods of follow-up  Case-control study—Give the eligibility criteria, and the sources and methods of case ascertainment and control selection. Give the rationale for the choice of cases and controls  Cross-sectional study—Give the eligibility criteria, and the sources and methods of selection of participants | 7 | Section: Recruitment and selection of study subjects |
|  |  | (b) Cohort study—For matched studies, give matching criteria and number of exposed and unexposed  Case-control study—For matched studies, give matching criteria and the number of controls per case | - |  |
| Variables | 7 | Clearly define all outcomes, exposures, predictors, potential confounders, and effect modifiers. Give diagnostic criteria, if applicable | 7-8 | Sections: Study outcomes, Measurements and questionnaire |
| Data sources/ measurement | 8* | For each variable of interest, give sources of data and details of methods of assessment (measurement). Describe comparability of assessment methods if there is more than one group | 7-8 | Sections: Measurements and questionnaire, Handling of data |
| Bias | 9 | Describe any efforts to address potential sources of bias | 7-9 |  |
| Study size | 10 | Explain how the study size was arrived at | 7-8, Figure 1 | Sections: Recruitment and selection of study subjects,  Measurements and questionnaire: ‘The questionnaire obtained conditional routing guidance’ |

Continued on next page

| Quantitative variables | 11 | Explain how quantitative variables were handled in the analyses. If applicable, describe which groupings were chosen and why | 7-9, Appendix: Table A1 | Sections: Measurements and questionnaire, Handling of data, Statistical analysis |
| --- | --- | --- | --- | --- |
| Statistical methods | 12 | (a) Describe all statistical methods, including those used to control for confounding | 9 | Statistical analysis section |
|  |  | (b) Describe any methods used to examine subgroups and interactions | 9 | Statistical analysis section: ‘Variance Inflation Factor and tolerance values indicated no multicollinearity… Analyses were based on complete cases.’ |
|  |  | (c) Explain how missing data were addressed | 9 | Handling of data section: ‘Missing values were excluded’ |
|  |  | (d) Cohort study—If applicable, explain how loss to follow-up was addressed  Case-control study—If applicable, explain how matching of cases and controls was addressed  Cross-sectional study—If applicable, describe analytical methods taking account of sampling strategy | - |  |
|  |  | (e) Describe any sensitivity analyses | 9 | Statistical analysis section |
| Results | | | | |
| Participants | 13* | (a) Report numbers of individuals at each stage of study—eg numbers potentially eligible, examined for eligibility, confirmed eligible, included in the study, completing follow-up, and analysed | 9, Figure 1 | Section: Results |
|  |  | (b) Give reasons for non-participation at each stage | 9, Figure 1 | Section: Results, Subsections: Prior experience with PNLC, No prior experience with PNLC |
|  |  | (c) Consider use of a flow diagram | Figure 1 |  |
| Descriptive data | 14* | (a) Give characteristics of study participants (eg demographic, clinical, social) and information on exposures and potential confounders | 9 | Section: Results |
|  |  | (b) Indicate number of participants with missing data for each variable of interest | Table 1 |  |
|  |  | (c) Cohort study—Summarise follow-up time (eg, average and total amount) |  |  |
| Outcome data | 15* | Cohort study—Report numbers of outcome events or summary measures over time |  |  |
|  |  | Case-control study—Report numbers in each exposure category, or summary measures of exposure |  |  |
|  |  | Cross-sectional study—Report numbers of outcome events or summary measures | 9-11; Figure 2-4 | Results: Subsection: Attitudes towards PNLC, Logistic Regression Analyses- Relevant predictors for PNLC |
| Main results | 16 | (a) Give unadjusted estimates and, if applicable, confounder-adjusted estimates and their precision (eg, 95% confidence interval). Make clear which confounders were adjusted for and why they were included | 11, Figure 4,  Page 9 | Logistic Regression Analysis- Relevant predictors for PNLC  Statistical analyses |
|  |  | (b) Report category boundaries when continuous variables were categorized | 10, Table 1 | Subsection: Participants characteristics, Variable: Age |
|  |  | (c) If relevant, consider translating estimates of relative risk into absolute risk for a meaningful time period | - |  |

*Give information separately for cases and controls in case-control studies and, if applicable, for exposed and unexposed groups in cohort and cross-sectional studies.

Note: An Explanation and Elaboration article discusses each checklist item and gives methodological background and published examples of transparent reporting. The STROBE checklist is best used in conjunction with this article (freely available on the Web sites of PLoS Medicine at http://www.plosmedicine.org/, Annals of Internal Medicine at http://www.annals.org/, and Epidemiology at http://www.epidem.com/). Information on the STROBE Initiative is available at www.strobe-statement.org.

**Study Questionnaire**

Dear participants,

The healthcare sector is undergoing change. Among other things, consideration is being given to adapting the roles of the various healthcare professions by expanding their responsibilities. This also involves testing models that have been successful in other European countries here in Germany.

In this questionnaire, we would like to ask for your opinion on whether certain tasks currently performed by GPs could also be carried out by other professional groups within a GP practice. This includes all medical professions working alongside GPs in GP practices: medical assistants (abbreviated to MFA), non-medical practice assistants and care assistants in GP practices (specially trained MFA, nursing staff, physician assistants and qualified nursing professionals. For practical reasons, we have grouped these professions under the term ‘PN’ in our questionnaire.

This questionnaire is aimed at patients who have a GP.

It will take approximately 10 minutes to complete.

**Thank you very well for your participation!**

| 1.) Do you have a GP | |
| --- | --- |
| ☐ Yes | ☐ No |

**Thank you very much for taking part in the survey!**

**2.)** How often have you visited your GP’s practice in the last 12 months?

☐ not at all

☐ 1-2 times

☐ 3-5 times

☐ more than 5 times

**3.)** How far do you commute to your GP practice?

_________ minutes **or** _________ hours

| **4.)** Are there any GP practices that are closer to or the same distance from your residency? | |
| --- | --- |
| ☐ Yes | ☐ No |

| **5.)** Do you have the impression that your GP practice is overburdened? | |
| --- | --- |
| ☐ Yes | ☐ No |

**6.) Please check your answer:**

|  | Completely agree | Rather agree | Neither agree nor disagree | Rather disagree | Completely disagree | I don’t know |
| --- | --- | --- | --- | --- | --- | --- |
| I am, in principle, in favour of certain tasks that were previously reserved for GPs being carried out by specially trained PNs. | **☐** | **☐** | **☐** | **☐** | **☐** | **☐** |

**7.)** **How do you currently view the PNs at your GP practice today?**

Please check the box to indicate to what extent you **agree or disagree** with the statements.

(Note: By ‘PN’, we refer to: medical assistants (abbreviated to MFA, colloquially known as ‘doctor’s assistants’), non-medical practice assistants and care assistants in GP practices (specially trained medical assistants), registered nurses, physician assistants and qualified nursing professionals)

|  | Completely agree | Rather agree | Neither agree nor disagree | Rather disagree | Completely disagree | I don’t know |
| --- | --- | --- | --- | --- | --- | --- |
| The PNs at my GP practice know how to engage with patients in an empathetic conversation about their condition(s). | **☐** | **☐** | **☐** | **☐** | **☐** | **☐** |
| I have complete confidence in the professional skills of the PNs at my GP practice. | **☐** | **☐** | **☐** | **☐** | **☐** | **☐** |
| I have complete confidence in my GP. | **☐** | **☐** | **☐** | **☐** | **☐** | **☐** |
| I’m in good hands at my GP practice. | **☐** | **☐** | **☐** | **☐** | **☐** | **☐** |

**8.)** In other countries, such as Denmark, the Netherlands and the UK, it is common practice for specially trained nurses, known as ‘advanced practice nurses’, to advise patients with specific chronic conditions during dedicated consultation hours. This includes, for example, patients with high blood pressure or diabetes mellitus. These “advanced practice nurses” have undergone specialist training through a degree course, often followed by further clinical training.

**With the following questions, we would like to find out to what extent you, as a patient, could envisage such a concept in GP practices in Germany in the future.** We would like to record your input as a patient, your views and ideas relating to this future concept. All assumptions are purely hypothetical.

Please imagine the following scenario: In your GP practice, specially trained PNs independently care for and advise patients during a consultation. These PN-led consultations conducted relate to conditions for which the PNs have received specialised training. The exact nature of this training is currently still the subject of academic debate.

**Please check your answer:**

|  | Already takes place | Would be willing | Would be somewhat willing | Would be somewhat unwilling | Would not be willing |
| --- | --- | --- | --- | --- | --- |
| Would you be willing to be treated by an PN during a consultation, even without your GP being present? | **☐** | **☐** | **☐** | **☐** | **☐** |

Please continue with **question 10 (page 4)**.

Please continue with **question 9** **(page 4)**.

**9.) Please indicate why you would reject / somewhat reject PN-led consultations:**

|  | Completely agree | Rather agree | Neither agree nor disagree | Rather disagree | Completely disagree | I don’t know |
| --- | --- | --- | --- | --- | --- | --- |
| I generally reject PN-led consultations. | **☐** | **☐** | **☐** | **☐** | **☐** | **☐** |
| Consultations are exclusively medical responsibilities performed by physicians. | **☐** | **☐** | **☐** | **☐** | **☐** | **☐** |
| I reject PN-led consultations because I would prefer to see only my GP. | **☐** | **☐** | **☐** | **☐** | **☐** | **☐** |
| Other reasons for rejection:  ____________________________________________________________________________________  ____________________________________________________________________________________ | | | | | | |

**Please continue with question 12 (page 6).**

**10.)** Please indicate the extent to which you **agree or disagree**.

| I think that PN-led consultations, compared to a GP-led consultations… | | | | | | |
| --- | --- | --- | --- | --- | --- | --- |
|  | Completely agree | Rather agree | Neither agree nor disagree | Rather disagree | Completely disagree | I don’t know |
| … would result in more time being available for my treatment. | **☐** | **☐** | **☐** | **☐** | **☐** | **☐** |
| … would enable me to gain a better understanding of my health problems. | **☐** | **☐** | **☐** | **☐** | **☐** | **☐** |
| … would lead to an earlier appointment in my general practice. | **☐** | **☐** | **☐** | **☐** | **☐** | **☐** |
| … would lead to a more regular intake of my medication. | **☐** | **☐** | **☐** | **☐** | **☐** | **☐** |
| … would make it easier for me to discuss difficulties with my medication. | **☐** | **☐** | **☐** | **☐** | **☐** | **☐** |
| I would agree to PN-led consultations without the GP being present… | | | | | | |
|  | Completely agree | Rather agree | Neither agree nor disagree | Rather disagree | Completely disagree | I don’t know |
| … to discuss my symptoms with me. | **☐** | **☐** | **☐** | **☐** | **☐** | **☐** |
| … to discuss possible side effects of the medication with me. | **☐** | **☐** | **☐** | **☐** | **☐** | **☐** |

**11.)** The next section focuses on specific diseases and health conditions, where we would like to know whether PN-led consultations would be an option for you.

**Please indicate whether you agree:**

Would you be willing, for the following medical issues, to be treated in a PN-led consultation by specially trained PNs, even without the GP being present?

*Please answer all sub-items, even if you do not suffer from the respective condition.*

|  | Already takes place | Would be willing | Would be somewhat  willing | Neither agree nor disagree | Would be somewhat unwilling | Would not be willing | Don’t know | |
| --- | --- | --- | --- | --- | --- | --- | --- | --- |
| Arterial hypertension | **☐** | **☐** | **☐** | **☐** | **☐** | **☐** | **☐** | |
| Follow-up examinations for diabetes mellitus | **☐** | **☐** | **☐** | **☐** | **☐** | **☐** | **☐** | |
| Follow-up examinations for bronchial asthma | **☐** | **☐** | **☐** | **☐** | **☐** | **☐** | **☐** | |
| Follow-up consultations for chronic obstructive pulmonary disease (COPD) | **☐** | **☐** | **☐** | **☐** | **☐** | **☐** | **☐** | |
| Superficial injuries e.g. abrasions | **☐** | **☐** | **☐** | **☐** | **☐** | **☐** | **☐** | |
| Chronic wounds such as leg ulcers | **☐** | **☐** | **☐** | **☐** | **☐** | **☐** | **☐** | |
| Certain thyroid disorders, e.g. hypothyroidism | **☐** | **☐** | **☐** | **☐** | **☐** | **☐** | **☐** |  |
| Acute conditions | | | | | | | |  |
| Gastrointestinal infections | **☐** | **☐** | **☐** | **☐** | **☐** | **☐** | **☐** |  |
| Urinary tract infections | **☐** | **☐** | **☐** | **☐** | **☐** | **☐** | **☐** |  |
| Upper respiratory tract infections | **☐** | **☐** | **☐** | **☐** | **☐** | **☐** | **☐** |  |
| New-onset backpain | **☐** | **☐** | **☐** | **☐** | **☐** | **☐** | **☐** |  |

| 12.) Do you have at least one chronic condition that requires the long-term use of at least one medication or the regular use of at least one medication or the regular use of an ointment or creme? (Note: contraceptives for women, such as „the pill “, are not included.) | |
| --- | --- |
| ☐ Yes | ☐ No |

Please continue with **question 18 (page 9)**.

| **13.)** Has at least one of these medications, ointments or creams been prescibed by your GP? | |
| --- | --- |
| ☐ Yes | ☐ No |

Please continue with **question 18 (page 9)**.

**14.)** In the following section, we would like to know whether, within the context of this future scenario, you could envisage **specially trained PNs adjusting the dosage of your long-term medication**, even without the need to contact your GP.

Long-term medication refers to medication that you have been taking daily for many weeks or years. Adjusting the dose means that the PNs would be permitted to increase or decrease the number of tablets of a long-term medication. The PNs would have received appropriate training and possess the necessary skills to conduct independent consultations involving dose adjustments for long-term medication. The exact nature of this training is currently still the subject of academic debate. The discontinuation or initiation of medication will continue to be carried out exclusively by your GP. Your GP will also continue to sign the prescriptions. Accordingly, the PNs would neither prescribe new medication nor replace an existing medication with a new one.

**Please check your answer:**

|  | Already takes place | Would be willing | Would somewhat be willing | Would somewhat be unwilling | Would not be willing |
| --- | --- | --- | --- | --- | --- |
| Would you be willing to have your long-term medication adjusted by specially trained PNs? | **☐** | **☐** | **☐** | **☐** | **☐** |

Please continue with **question 15** **(page 7)**.

Please continue with **question 16** **(page 7)**.

**15.) Please explain why you reject dosage adjusting of your long-term medication by PNs:**

|  | Completely agree | Rather agree | Neither agree nor disagree | Rather disagree | Completely disagree | I don’t know |
| --- | --- | --- | --- | --- | --- | --- |
| I am generally opposed to changes in the dosage of long-term medication by PNs. | **☐** | **☐** | **☐** | **☐** | **☐** | **☐** |
| Adjustments to the dosage of long-term medication are exclusively general practitioners’ procedures | **☐** | **☐** | **☐** | **☐** | **☐** | **☐** |
| Other reasons of rejection:  _____________________________________________________________________________________  _____________________________________________________________________________________ | | | | | | |

Please continue with **question** **18 (page 9)**.

**16.)** Would you be willing to have the dosage of the following medicines adjusted by a specially trained PNs, even without your GP being present?

*Please answer this question even if you are not taking these medicines.*

|  | Already takes place | Would be willing | Would be somewhat willing | Neither agree nor disagree | Would be somewhat unwilling | Would not be willing | Don’t know |
| --- | --- | --- | --- | --- | --- | --- | --- |
| Antihypertensives | **☐** | **☐** | **☐** | **☐** | **☐** | **☐** | **☐** |
| Oral antidiabetic drugs | **☐** | **☐** | **☐** | **☐** | **☐** | **☐** | **☐** |
| Injectable diabetes medications (e.g. insulin) | **☐** | **☐** | **☐** | **☐** | **☐** | **☐** | **☐** |
| Thyroid medication | **☐** | **☐** | **☐** | **☐** | **☐** | **☐** | **☐** |
| Asthma medication (e.g. inhalers) | **☐** | **☐** | **☐** | **☐** | **☐** | **☐** | **☐** |
| COPD medication (e.g. inhalers) | **☐** | **☐** | **☐** | **☐** | **☐** | **☐** | **☐** |
| Cholesterol-lowering medication | **☐** | **☐** | **☐** | **☐** | **☐** | **☐** | **☐** |
| Painkillers | **☐** | **☐** | **☐** | **☐** | **☐** | **☐** | **☐** |
| Over-the-counter medication | **☐** | **☐** | **☐** | **☐** | **☐** | **☐** | **☐** |
| Blood-thinning medication (e.g. Falithrom) | **☐** | **☐** | **☐** | **☐** | **☐** | **☐** | **☐** |
| In addition, for the following medicines: ____________________________________________________________________________________ | | | | | | | |

**17.)** Below are some possible statements regarding the framework conditions for **consultations and dose adjustments for long-term medication by PNs.** We would like to know which points you personally consider to be important.

| I would consider it important, … | | | | | | |
| --- | --- | --- | --- | --- | --- | --- |
|  | Completely agree | Rather agree | Neither agree nor disagree | Rather disagree | Completely disagree | I don’t know |
| … to know in advance whom, I will see (either the PN or the GP). | **☐** | **☐** | **☐** | **☐** | **☐** | **☐** |
| … that my GP discusses with me in advance that the medical assistants will be taking on additional duties. | **☐** | **☐** | **☐** | **☐** | **☐** | **☐** |
| … that I see the GP at every second appointment. | **☐** | **☐** | **☐** | **☐** | **☐** | **☐** |
| … that the PN consults with my GP at the end of the day. | **☐** | **☐** | **☐** | **☐** | **☐** | **☐** |
| …that the PN consults with my GP after consultation. | **☐** | **☐** | **☐** | **☐** | **☐** | **☐** |
| … that I can choose whether I have an appointment with my GP or with the PN. | **☐** | **☐** | **☐** | **☐** | **☐** | **☐** |
| … that I can involve my GP at any time if I have uncertainties. | **☐** | **☐** | **☐** | **☐** | **☐** | **☐** |

**18) Finally, a few questions about yourself**

How long have you been a patient at your current GP practice?

________________	years

If you have been a patient at your current GP practice for less than one year, please state the length of time in months below: ____________________________________

**☐** I don’t know.

| Have you ever been medically diagnosed with any of the following chronic conditions? | Yes | No |
| --- | --- | --- |
| Asthma, including allergic asthma | **☐** | **☐** |
| Chronic bronchitis, chronic obstructive pulmonary disease, pulmonary emphysema | **☐** | **☐** |
| Heart attack | **☐** | **☐** |
| Coronary artery disease | **☐** | **☐** |
| Stroke | **☐** | **☐** |
| Heart insufficiency | **☐** | **☐** |
| Chronic back pain | **☐** | **☐** |
| Diabetes mellitus (excluding gestational diabetes) | **☐** | **☐** |
| High blood pressure | **☐** | **☐** |
| Thyroid disorders, e.g. hypothyroidism | **☐** | **☐** |
| Others: ______________________________________ | **☐** | **☐** |

**Now there are just a few final questions we need to ask in order to better assess the survey results.**

How many GPs work at your GP practice?

**☐** 1 GP

**☐** More than 1 GP

How many people live in **your town?**

**☐** Under 2,000 inhabitants

**☐** 2,001 to 5,000 inhabitants

**☐** 5,001 to 50,000 inhabitants

**☐** 50,001 to 100,000 inhabitants

**☐** 100,001 to 500,000 inhabitants

**☐** 500,001 or more inhabitants

***Thank you very much for taking the time to complete this questionnaire!***
